# Quercetin Attenuates Inflammatory Responses in BV-2 Microglial Cells: Role of MAPKs on the Nrf2 Pathway and Induction of Heme Oxygenase-1

**DOI:** 10.1371/journal.pone.0141509

**Published:** 2015-10-27

**Authors:** Grace Y. Sun, Zihong Chen, Kimberly J. Jasmer, Dennis Y. Chuang, Zezong Gu, Mark Hannink, Agnes Simonyi

**Affiliations:** 1 Biochemistry Department, University of Missouri, Columbia, Missouri, United States of America; 2 Division of Biological Sciences, University of Missouri, Columbia, Missouri, United States of America; 3 Interdisciplinary Neuroscience Program, University of Missouri, Columbia, Missouri, United States of America; 4 Department of Pathology and Anatomical Sciences, University of Missouri, Columbia, Missouri, United States of America; 5 Center for Botanical Interaction Studies, University of Missouri, Columbia, Missouri, United States of America; Indiana School of Medicine, UNITED STATES

## Abstract

A large group of flavonoids found in fruits and vegetables have been suggested to elicit health benefits due mainly to their anti-oxidative and anti-inflammatory properties. Recent studies with immune cells have demonstrated inhibition of these inflammatory responses through down-regulation of the pro-inflammatory pathway involving NF-κB and up-regulation of the anti-oxidative pathway involving Nrf2. In the present study, the murine BV-2 microglial cells were used to compare anti-inflammatory activity of quercetin and cyanidin, two flavonoids differing by their alpha, beta keto carbonyl group. Quercetin was 10 folds more potent than cyanidin in inhibition of lipopolysaccharide (LPS)-induced NO production as well as stimulation of Nrf2-induced heme-oxygenase-1 (HO-1) protein expression. In addition, quercetin demonstrated enhanced ability to stimulate HO-1 protein expression when cells were treated with LPS. In an attempt to unveil mechanism(s) for quercetin to enhance Nrf2/HO-1 activity under endotoxic stress, results pointed to an increase in phospho-p38MAPK expression upon addition of quercetin to LPS. In addition, pharmacological inhibitors for phospho-p38MAPK and MEK1/2 for ERK1/2 further showed that these MAPKs target different sites of the Nrf2 pathway that regulates HO-1 expression. However, inhibition of LPS-induced NO by quercetin was not fully reversed by TinPPIX, a specific inhibitor for HO-1 activity. Taken together, results suggest an important role of quercetin to regulate inflammatory responses in microglial cells and its ability to upregulate HO-1 against endotoxic stress through involvement of MAPKs.

## Introduction

Recent studies have demonstrated ability for a number of botanical polyphenols to suppress oxidative stress and inflammatory responses in immune cells [1–5]. In many instances, microglial cells were used as a model to test effects of the botanical compounds on induction of pro-inflammatory genes by lipopolysaccharide (endotoxin, LPS), which activates toll-like receptors and stimulates signaling pathways involving NF-κB [6]. Recognition for botanical phenolics to mitigate LPS-induced nitrosative/oxidative stress underscores the important role of these compounds as potential therapeutic targets to ameliorate neurodegenerative diseases [7].

Studies on nitrosative/oxidative stress and inflammatory responses in neurodegenerative diseases have also indicated the importance of the anti-oxidant pathway involving the Kelch-like ECH-associated protein 1 (Keap1)/Nuclear Factor Erythroid 2-Like 2 (NFE2L2, Nrf2). This pathway is responsible for transcriptional activation of a large number of genes that are regulated by Anti-oxidant Response Elements (AREs) in their promoters. Many Nrf2-dependent genes encode anti-oxidant proteins, which enable cells to counteract nitrosative/oxidative stress and restore redox homeostasis [8,9]. In cells cultured under homeostatic conditions, steady-state levels of Nrf2 are kept low because Keap1, which serves as an adaptor protein for an E3 ubiquitin ligase, targets Nrf2 for ubiquitin-dependent degradation by the proteasome [10–12]. There is strong evidence that structurally diverse compounds produced from within the cell as well as from exogenous sources (including a number of plant polyphenols) can perturb Keap1-mediated repression of Nrf2, leading to increased levels of Nrf2 and subsequent transcriptional activation of Nrf2-dependent genes [13]. Special attention has been drawn to the beneficial aspects of Nrf2-mediated induction of heme oxygenase 1 (HO-1), the enzyme that degrades heme to generate CO, biliverdin and free iron [14]. These studies have demonstrated an important role for HO-1 in immunoregulation, oxidative stress, and resistance to bacterial infection [14–17].

Many fruits and vegetables contain high levels of flavonoids with anti-oxidant properties and are thus suggested to elicit health benefits through their ability to modulate redox homeostasis [7,18]. Among these, quercetin, a flavonol enriched in berries, apples and red onion, has been shown to offer special health benefits including protective effects in both central and peripheral systems [19–23]. The ability of dietary phytochemicals to modulate cellular stress responses suggests new modalities for therapeutic intervention in neurodegenerative disorders [24,25].

Recent studies have attributed the anti-oxidative/anti-nitrosative and anti-inflammatory effects of quercetin to its ability to inhibit the NF-κB pathway and to up-regulate the Nrf2 transcriptional pathway [5,26,27]. However, the mechanism(s) and the extent for this flavonol to modulate these pathways remain elusive. In the present study, we investigated the ability for quercetin and cyanidin, a structurally similar flavonoid, to inhibit LPS-induced NO and to stimulate the Nrf2 pathway that leads to increased HO-1 expression in BV-2 microglial cells. Our study was further directed to the effects of MAPKs, in particular, ERK1/2 and p38MAPK, which have been implicated in regulation of both NF-κB and Nrf2 pathways.

## Materials and Methods

### Materials

Lipopolysaccharide (LPS) (rough strains) from Escherichia coli F583 (Rd mutant) and fetal bovine serum (FBS) were obtained from Sigma-Aldrich (St. Louis, MO). FBS (lot no. 12J002) was qualified USA origin, sterile, filtered and cell culture tested, and certified to contain endotoxin levels less than 1.0 ng/ml. Dulbecco's modified Eagle's medium (DMEM), penicillin, streptomycin, 0.05% (w/v) trypsin/EDTA, and phosphate-buffered saline (PBS) were obtained from GIBCO (Gaithersburg, MD). Tin Protoporphyrin IX dichloride (TinPPIX) was from Santa Cruz Biotechnology (Santa Cruz, CA). Kinase inhibitors (U0126 and SB202190) were from Cell Signaling (Beverly, MA).

Antibodies used for Western blots include: goat anti-rabbit IgG-horseradish peroxidase, goat anti-mouse IgG-horseradish peroxidase, rabbit polyclonal anti-Nrf2, and anti-HO-1 (Santa Cruz Biotechnology, Santa Cruz, CA); mouse monoclonal anti-β-actin (Sigma-Aldrich, St. Louis, MO); rabbit polyclonal anti-ERK1/2, mouse monoclonal anti-phospho-ERK1/2, rabbit monoclonal anti-p38MAPK, and anti-phospho-p38MAPK (Cell Signaling, Beverly, MA). Quercetin (Sigma-Aldrich, St. Louis, MO) and cyanidin chloride (Indofine Chemical Comp., Hillsborough, NJ) were dissolved in DMSO as a stock solution.

### Cell culture and treatments

The murine BV-2 cell line was generated by infecting primary microglia cell cultures with a v-raf/v-myc oncogene carrying retrovirus (J2) [28]. These cells were obtained as a gift from Dr. R. Donato [29] and prepared as previously described [30]. Cells were cultured in 75 cm^2^ flasks with DMEM supplemented with 5% FBS containing 100 units/ml penicillin and100 μg/ml streptomycin, and maintained in 5% CO_2_ incubator at 37°C. Normally, BV-2 cells were grown in 100 mm dish and after 80–90% confluent, they were removed and subcultured in 6-, 12-, or 96-well plates, depending on the experiment. Cells were then cultured overnight or until 80–90% confluent. Cells were serum starved for 3 h followed by adding botanical compounds (quercetin 0–20 μM or cyanidin, 0–100 μM) or enzyme inhibitors (0–10 μM) for 1 h and then stimulated with LPS (0–200 ng/ml). Test compounds were dissolved in DMSO and added to culture at concentrations of less than 0.5%. In most experiments, cell culture was tested to ensure the levels of DMSO exerted no deleterious effects on the responses. In some studies, cell morphology was examined using a phase contrast Nikon DIAPHOT 300 microscope attached with a CCD cool camera linked to the MagnaFire 2.1C software for image processing.

### Assessment of cell viability

The WST-1 protocol was used for assessment of cell viability. Briefly, after reaching 80–90% confluency, cells in 96-well plates were serum starved for 3 h, followed by treatment with botanical compounds or inhibitors for 1 h and then stimulated with LPS for 16 h. After treatment, aliquots of the culture medium were taken for assay of nitric oxide (NO) and the remaining culture was used for cell viability assay by adding 10 μl of the WST-1 reagent (Roche, Mannheim, Germany). After gentle shaking, cells were incubated for 2 h at 37°C and absorbance was read at 420–480 nm (with reference at >600 nm).

### Nitric oxide determination in culture medium

NO released from cells was converted to nitrite in the culture medium, which was determined using the Griess reagent [1,31]. Cells were cultured in DMEM without phenol red. Sixteen hours after LPS treatment, aliquots (50 μl) of culture medium were transferred to 96-well plates and incubated with 50 μl of reagent A [1% (w/v) sulfanilamide (Sigma-Aldrich, St. Louis, MO) in 5% phosphoric acid] for 10 minutes at room temperature in the dark. Incubation with 50 μl of reagent B (0.1%, w/v, N-1-napthylethylenediamine dihydrochloride, Sigma-Aldrich, St. Louis, MO) for 10 minutes at room temperature in the dark was followed by measurement of the absorbance at 543 nm using the Synergy-4 plate reader. Sodium nitrite (0–100 μM), serially diluted in culture media, was used to prepare the nitrite standard curve.

### Western blot analysis

Protocol for Western blot analysis was similar to that described by Jiang et al. [2]. Briefly, cells were harvested in Laemmli buffer and centrifuged at 10,000 × *g* for 15 minutes at 4°C and transferred to a clean tube to remove cell debris. The supernatant was collected in Eppendorf tubes and frozen at -80°C until use. Equal amount of protein was loaded and resolved in SDS-PAGE and run at 100 V. After electrophoresis, proteins were transferred to 0.45 μm nitrocellulose membranes at 100 V for 1.5 h. Membranes were blocked in Tris-buffered saline (TBS), pH 7.4, with 0.1% Tween 20 (TBS-T) containing 5% non-fat milk for 1.5 h at room temperature. For different experiments, the blots were incubated with anti-Nrf2 (1:500), anti-HO-1 (1:800), anti-ERK1/2 (1:1,000), anti-phospho-ERK1/2 (1:1,000), anti-p38MAPK (1:1,000), anti-phospho-p38MAPK (1:1,000) overnight at 4°C. After repeated washing with 1X TBS-T, blots were incubated with goat anti-rabbit IgG-HRP (1:6,000) or goat anti-mouse IgG-HRP (1:6,000) for 1 h at room temperature. The blots were then washed three times with 1X TBS-T. Immuno-labeling was detected by chemiluminescence ECL/WestPico/Femto and developed in X-ray film developer. For loading controls, blots were incubated with anti-β-actin (1:50,000). Films were scanned and the optical density of protein bands was measured using the QuantityOne software program (BioRad, Hercules, CA).

### RNA Isolation and quantitative PCR analysis

One day prior to RNA isolation, 1.2 x 10^6^ BV-2 cells were plated onto each of 2 wells of a 6-well plate. The following morning, the cells were serum-starved for 3 h with pretreatment with quercetin and inhibitors for 1 h followed by treatment with or without LPS (100 ng/ml) for 6 h. Immediately following the treatment, total RNA was isolated using the Qiagen RNeasy mini kit according to manufacturer’s instructions. For each treatment, the two replicate wells were combined for RNA isolation. Homogenization of the sample was achieved by passing the entire sample through an 18-guage needle, 10 times. The RNA was eluted in RNAse-free water and stored at -80°C overnight. Forty percent of the isolated RNA from each sample was used to synthesize cDNA, which was done using the High Capacity cDNA Reverse Transcription Kit (Applied Biosystems) according to manufacturer’s instructions. cDNA was purified using the GeneJet PCR purification kit.

Quantitative PCR was carried out on an Applied Biosystems® 7500 Real Time PCR System for 40 cycles. All PCR reactions were conducted in triplicate in an Applied Biosystems® MicroAmp® Optical 96-well Reaction Plate with a 25 μl reaction volume containing 12.5 μl of Thermo Scientific Maxima SYBR Green/ROX qPCR Master Mix, 7 μl of purified cDNA and a final primer concentration of 0.15 μM for both forward and reverse primers. The cDNA in each replicate represents approximately 5% of the total RNA collected, or 1 x 10^5^ cells. Primers were obtained from Sigma-Aldrich: HMOX1, 5’-GCACCGGCCGGATGGAGCGTCC-3’ and 5’-CGTCTCGGGTCACCTGGCCCTTCTG-3’, and Actin, 5’-CTTTGCCGATCCGCCGCCCGTCCACAC-3’ and 5’-GAGGGGAAGACGGCCCGGGGGGCATCGTC-3’. The threshold for detection of each mRNA of interest was reached between cycles 16 and 24. Expression levels were analyzed using the Comparative CT Method for quantitative RT-PCR. Briefly, expression is normalized to actin and then shown as the fold-change over an untreated sample.

### Statistical analysis

With exception of the RT-PCR experiments, data are shown as mean ± SEM from at least three independent experiments. Results were analyzed by either one-way or two-way ANOVA followed by Bonferroni post-tests (V4.00; GraphPad Prism Software Inc., San Diego, CA). Differences were considered significant at p<0.05 for all analyses. In the experiment involving RT-PCR, error was shown as the standard deviation of the triplicate PCR reactions and results of a representative experiment from 3–4 experiments were presented. Comparisons were carried out using simple Student’s t-test.

## Results

### Effects of quercetin and cyanidin on LPS-induced NO production in BV-2 microglial cells

**Fig 1:**
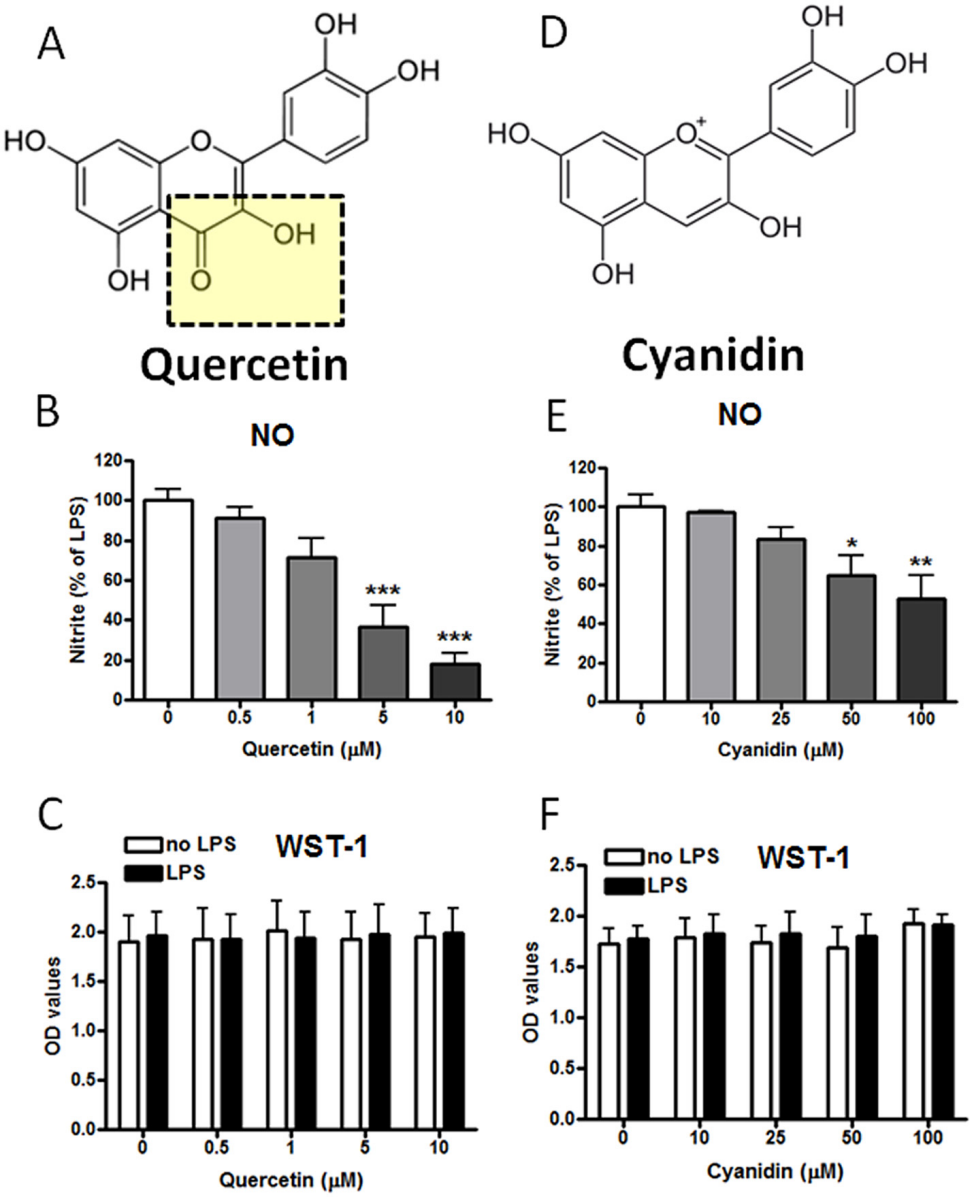
Effects of quercetin and cyanidin on cell viability and NO production induced by LPS in BV-2 microglial cells. (A, D) *Chemical structures of quercetin and cyanidin*. (B, E) *NO production*. Cells cultured in 96 well-plate were serum starved for 3 h followed by treating with quercetin or cyanidin for 1 h and followed by stimulation with LPS (100 ng/ml) for 16 h. Aliquots of the culture medium was removed for measurement of NO by the Griess protocol. Results are expressed as the mean ± SEM (n = 3–5) and significant difference from the LPS-stimulated group was determined by one-way ANOVA followed by Bonferroni post-tests. *p<0.05, **p<0.01, ***p < 0.001. (C, F) *Cell viability*. For assay of cell viability, after aliquots of the culture medium were taken for assay of NO, 10 μl of WST-1 reagent was added and incubated for 2 h. Absorbance was read at 420–480 nm. Results are expressed as the mean ± SEM from three independent experiments. Data were analyzed by two-way ANOVA and no significant effects were found.

Based on our recent study demonstrating different responses among the berry flavonoids to endotoxic stress [4], quercetin and cyanidin, two flavonoids with similar structure (Fig 1A and 1D) were selected to test their effects on LPS-induced NO production as well as cell viability. As shown in Fig 1B and 1E, while quercetin potently inhibited LPS-induced NO at 5 μM, a much higher concentration of cyanidin, up to 50 μM, was required to achieve significant inhibition of LPS-induced NO production. Using the WST-1 cell viability assay, neither compound had adverse effects on cell viability at the concentrations tested, either in the absence or the presence of LPS (Fig 1C and 1F).

### LPS induces Nrf2 and HO-1 protein expression in BV-2 microglial cells

**Fig 2:**
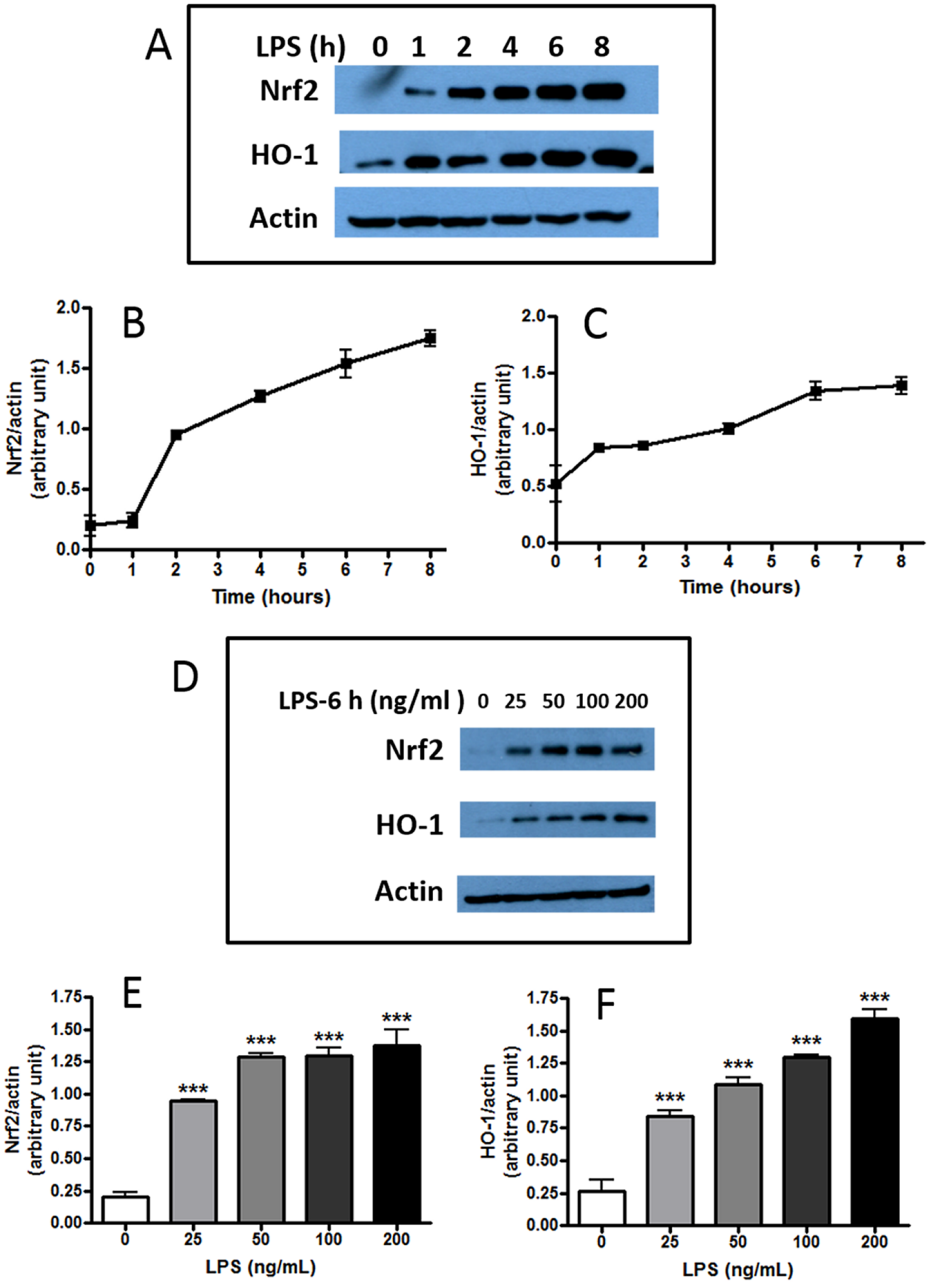
LPS induces Nrf2 and HO-1 protein expression in BV-2 microglial cells. Confluent cells were serum starved for 4 h prior to stimulation with LPS (100 ng/ml) and assay for Nrf2 and HO-1 expression at different times. (A, B, C). Time- dependent increase in Nrf2 and HO-1 protein expression after stimulation with LPS (100 ng/ml). Results are means ± SEM of three independent experiments. (D, E, F) Dose-dependent increase of Nrf2 and HO-1 protein expression by LPS after a 6 h incubation time. Results are means ± SEM of three independent experiments and were analyzed by one-way ANOVA followed by Bonferroni post-tests. ***p<0.001 vs. no LPS control.

Despite of the ability for LPS to stimulate the canonical NF-κB pathway and induce pro-inflammatory cytokines and NO production in microglial cells, there is evidence that this endotoxin itself also can stimulate the Nrf2 pathway and induce HO-1, albeit to a smaller extent [32–34]. To better define experimental conditions that would allow us to examine the ability of quercetin and cyanidin to modulate expression of Nrf2 and HO-1 in LPS-stimulated cells, we first characterized the ability of LPS to stimulate the Nrf2/HO-1 pathway in BV-2 microglial cells. As shown in Fig 2A, addition of LPS (100 ng/ml) to BV-2 cells resulted in a time-dependent increase in levels of both Nrf2 and HO-1 proteins over an 8 h time course (Fig 2B and 2C). Using a 6 h exposure time, levels of Nrf2 and HO-1 protein expression were measured following treatment with different concentrations of LPS (Fig 2D). Results show increased expression of both Nrf2 and HO-1 proteins in a dose-dependent manner up to 200 ng/ml (Fig 2E and 2F). In subsequent experiments, a dose of LPS at 100 ng/ml and an incubation time of 6 h were selected for studies.

### Effects of quercetin and cyanidin on Nrf2 pathway and HO-1 production in the presence and absence of LPS

**Fig 3:**
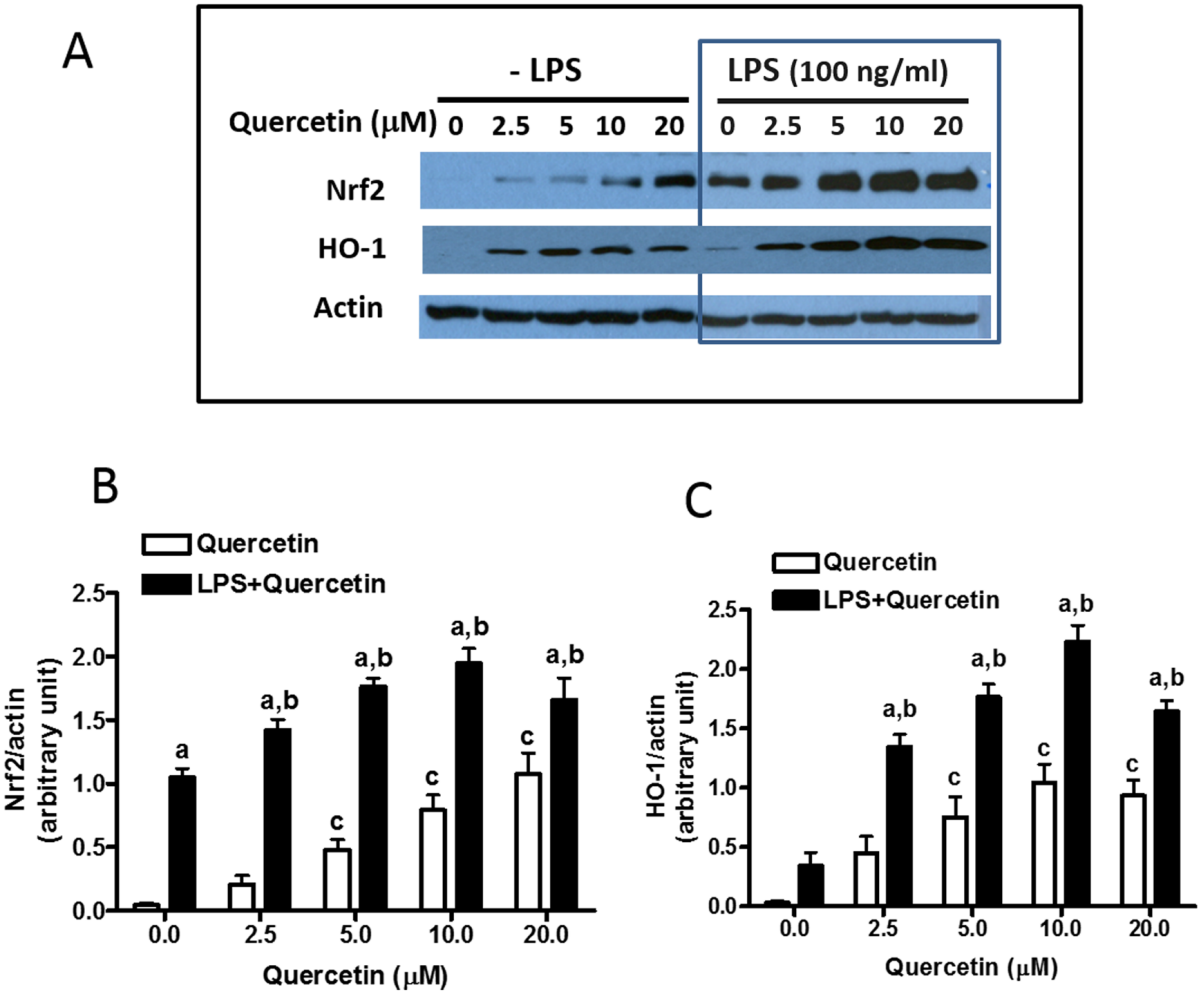
Effects of quercetin on induction of Nrf2 and HO-1 proteins in the presence and absence of LPS. BV-2 microglial cells were cultured in 12-well plate. After confluent, cells were serum starved for 3 h followed by adding quercetin at different concentrations for 1 h and followed by stimulation with LPS (100 ng/ml) for 6 h. (A) A representative blot from 3–5 experiments. (B, C) Bar graphs represent Nrf2/actin and HO-1/actin ratios. Results are expressed as the mean ± SEM (n = 3–5) and analyzed by two-way ANOVA with Bonferroni post-tests (see text for details). “a” denotes significant differences between LPS+quercetin vs. quercetin alone; “b” denotes significant differences between LPS+quercetin vs. LPS alone; “c” denotes significant differences as compared to 0 μM quercetin.

The difference between quercetin and cyanidin to inhibit LPS-induced NO production alerted us to examine their ability to stimulate the Nrf2 pathway for induction of HO-1. As shown in Fig 3, exposure of BV-2 cells to quercetin for 7 hours resulted in a dose-dependent increase in the levels of Nrf2 and HO-1 proteins (Fig 3A–3C). In the same experiment, BV-2 cells were pretreated with quercetin for 1 h prior to stimulation with LPS (100 ng/ml) for 6 h without removal of quercetin. Quercetin pretreatment resulted in a modest but statistically significant increase in Nrf2 expression after LPS exposure (Fig 3B). Data analysis by two-way ANOVA revealed a significant effect of LPS (p<0.0001) and quercetin (p<0.0001), and a significant interaction (p = 0.0180). Bonferroni post-test indicated significant differences between the quercetin and LPS+quercetin groups as well as the LPS and LPS+quercetin groups for each concentration of quercetin tested. For HO-1 protein expression, quercetin pretreatment also resulted in a statistically significant increase following LPS treatment, with maximum induction of HO-1 protein expression observed at 10 μM quercetin and 100 ng/ml LPS (Fig 3A and 3C). For example, a four-fold increase in HO-1 protein expression was observed in cells treated with LPS and quercetin at 2.5 μM over the level in cells treated LPS alone (Fig 3C). Data analysis by two-way ANOVA revealed a significant interaction (p = 0.0234) and main effects of both quercetin (p<0.0001) and LPS (p<0.0001). Bonferroni post-test indicated significant differences in HO-1 protein expression between the quercetin and LPS+quercetin groups as well as between the LPS and LPS+quercetin groups for each concentration. It is of interest to note that in our study, treating cells with quercetin at 20 μM prior to stimulation with LPS appeared to show Nrf2 and HO-1 levels lower than those treated with 10 μM (Fig 3). Although the reason for the decrease in expression is not understood, there was no apparent indication of cell death under this condition.

**Fig 4:**
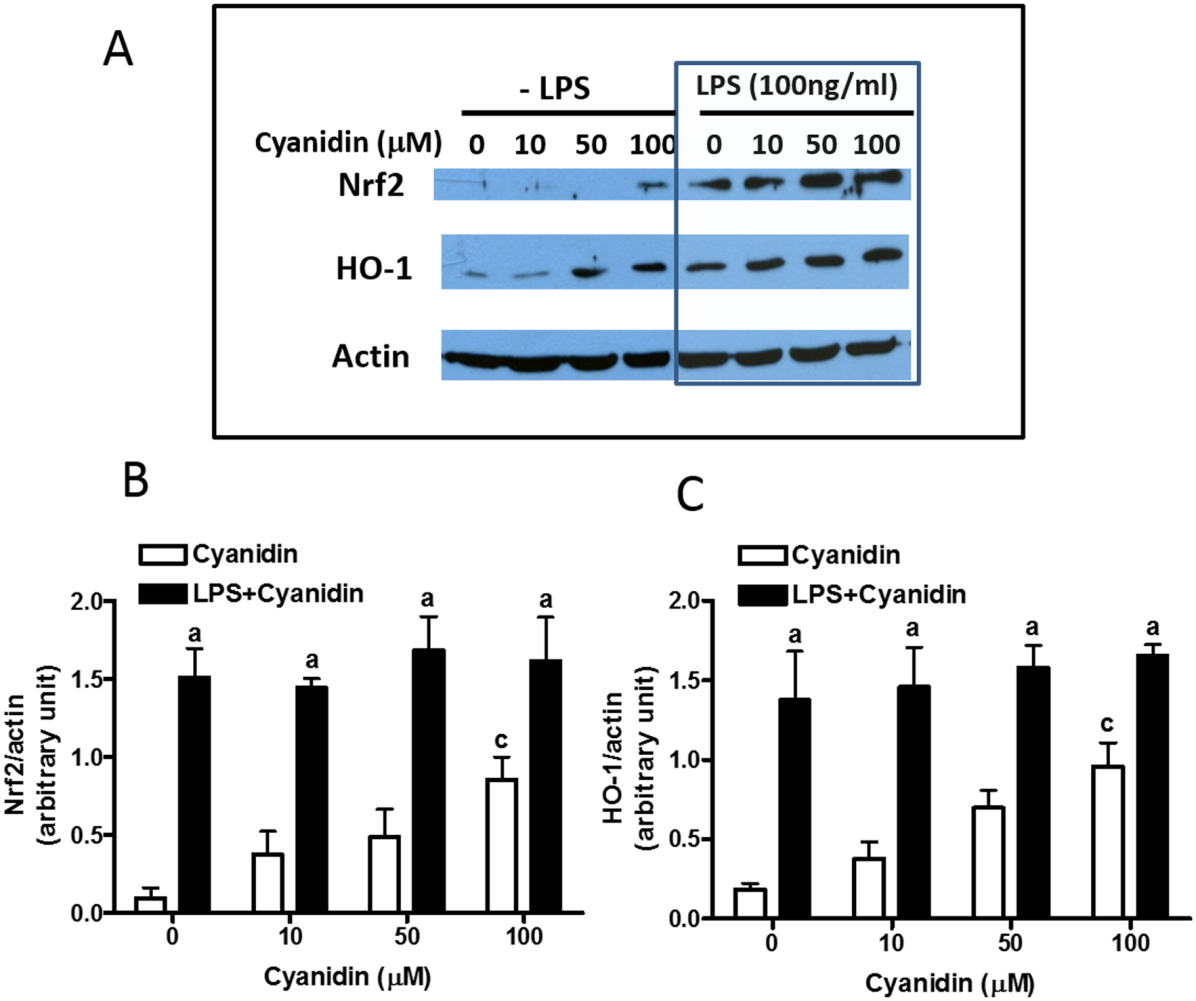
Effects of cyanidin on induction of Nrf2 and HO-1 proteins in the presence and absence of LPS. Protocol for treatment of cells is the same as described in Fig 4. (A) A representative blot from 3–4 experiments. (B, C) Bar graphs represent Nrf2/actin and HO-1/actin ratios. Results are expressed as the mean ± SEM (n = 3–4) and analyzed by two-way ANOVA with Bonferroni post-tests (see text for details). “a” denotes significant differences between LPS+cyanidin vs. cyanidin alone; “c” denotes significant differences as compared to 0 μM cyanidin.

In contrast to quercetin, cyanidin treatment resulted in a significant increase of Nrf2 and HO-1 protein levels only at the highest concentration of cyanidin used, 100 μM (p<0.05) (Fig 4A, 4B and 4C). Furthermore, cyanidin was not able to augment the expression of Nrf2 and HO-1 induced by LPS, as no significant interaction was obtained (p>0.05) (Fig 4).

### Quercetin enhances LPS-induced phospho-p38MAPK

**Fig 5:**
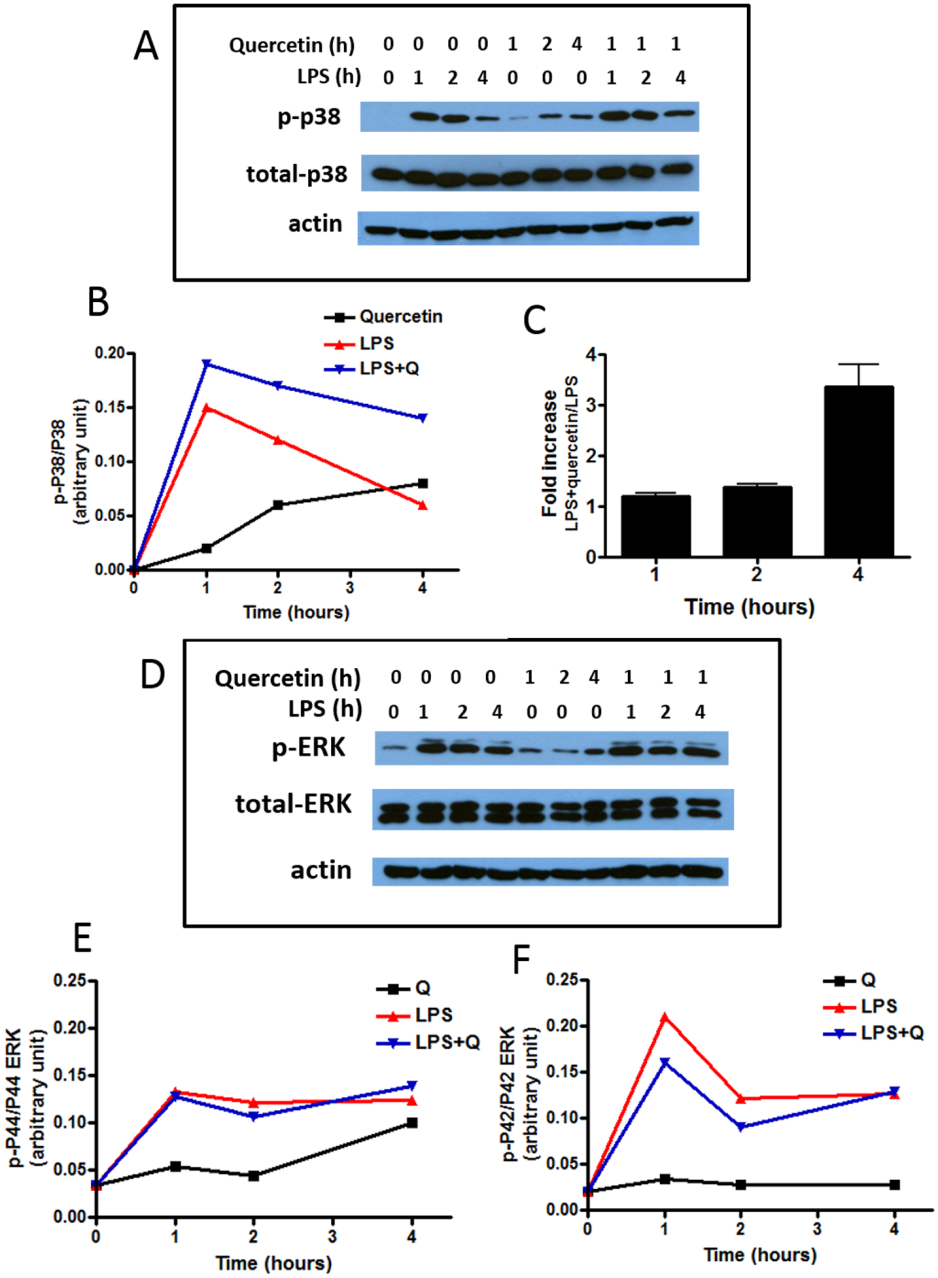
Effects of LPS and/or quercetin on phosphorylation of p38MAPK and ERK1/2. BV-2 microglial cells were treated with LPS (100 ng/ml), quercetin (Q, 12.5 μM), and LPS+quercetin for 1, 2 and 4 h. Samples were taken for Western blot analysis for (A) phospho- and total p38MAPK and (D) phospho- and total ERK1/2. Results denote one representative blot from four independent experiments and also shown as ratios on B, E, F. Bar graph 6C represents ratios of p-p38MAPK/p38MAPK for LPS+quercetin versus LPS from different time points (means ± SEM, n = 4).

A number of studies, including those from our own, have demonstrated the ability for LPS to stimulate the canonical NF-κB and MAPK pathways for induction of iNOS [1,5,31,35,36]. In order to test the effects of quercetin on the MAPK activities, BV-2 cells were separately treated with LPS (100 ng/ml), quercetin (12.5 μM), or with both LPS and quercetin, and levels of the phosphorylated forms of p38MAPK and of ERK1/2 were determined at 1, 2 and 4 h. As shown in Fig 5A and 5B, LPS treatment alone induced a rapid but transient induction of phosphorylated p38MAPK, while quercetin showed a smaller but sustained increase. Nevertheless, treatment with both quercetin and LPS resulted in higher levels of phosphorylated p38MAPK as compared to that with LPS alone, especially at the 4 h time point (Fig 5C). Treatment with LPS also stimulated phosphorylation of p42 and p44 isoforms of ERK (Fig 5D–5F). However, while quercetin resulted only in a modest increase in ERK1/2 activation, the combination of LPS and quercetin did not show a significant increase in p-ERK1/2 levels as compared to treatment with LPS alone (Fig 5E and 5F).

### Role of p38MAPK and ERK1/2 on quercetin-induced Nrf2 and HO-1 protein expression

**Fig 6:**
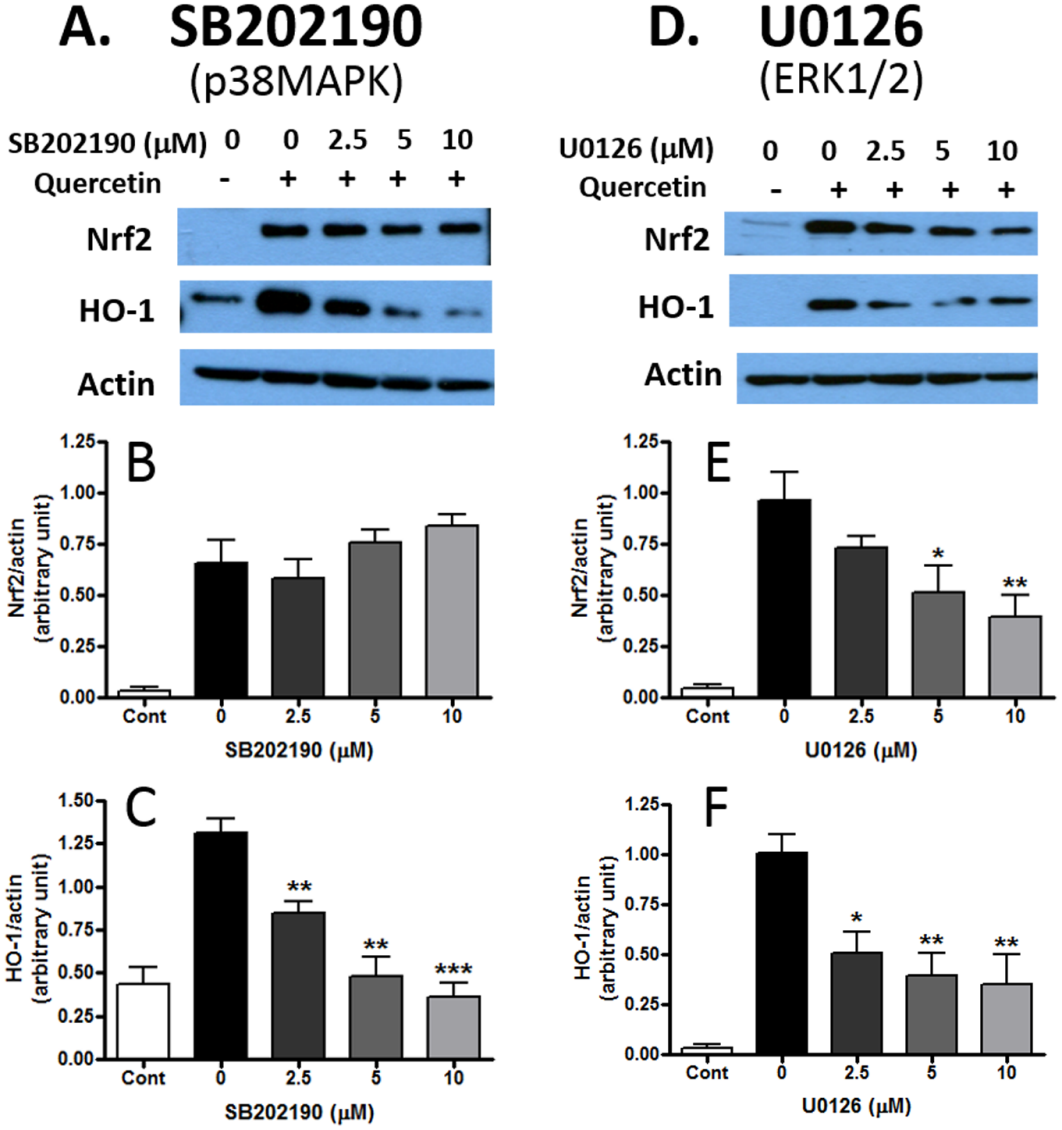
Effects of MAPK inhibitors on Nrf2 and HO-1 protein expression induced by quercetin. Cells were treated with quercetin (12.5μM, 7 h) together with different doses of (A, B, C) SB202190, inhibitor for p38MAPK, and (D, E, F) U0126, inhibitor for MEK1/2 and ERK1/2. Bar graphs represent Nrf2/actin and HO-1/actin ratios (means ± SEM of four independent experiments). Data were analyzed by one-way ANOVA followed by Bonferroni post-tests. *p<0.05, **p<0.01, ***p<0.001 vs. control (no quercetin, no inhibitor).

To determine if activation of either p38MAPK or ERK1/2 contributed to increased expression of Nrf2 and of HO-1 by quercetin, we used SB202190, a specific inhibitor of p38MAPK, and U0126, a specific inhibitor of MEK1/2, the kinases leading to phosphorylation of ERK1/2. As shown in Fig 6A–6C, SB202190 inhibited quercetin-induced HO-1 expression in a dose-dependent manner but induction of Nrf2 protein expression was not altered. On the other hand, U0126 significantly inhibited quercetin-induced Nrf2 protein expression as well as HO-1 protein expression (Fig 6D–6F). These results suggest that while ERK1/2 activity is required for stabilization of Nrf2 and subsequent activation of HO-1 transcription, p38MAPK activity is not required for stabilization of Nrf2 although it is required for induction of HO-1 protein expression.

### SB202190 inhibits quercetin-induced HMOX-1 mRNA expression

**Fig 7:**
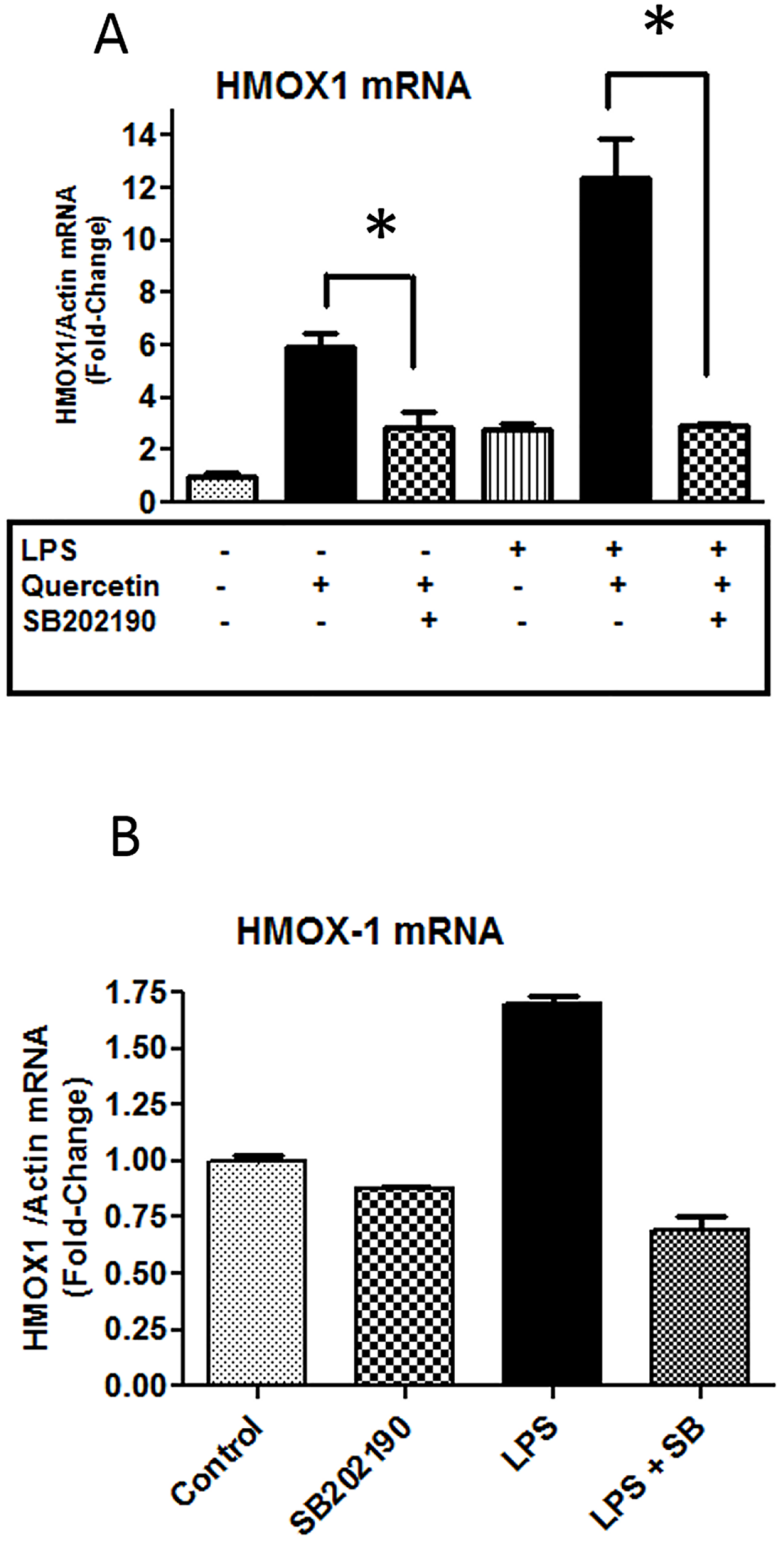
Effects of SB202190 on LPS and/or quercetin induced HMOX1 mRNA expression in BV-2 microglial cells. (A) Microglial cells were treated with quercetin (12.5 μM and SB202190 (10 μM) for 1 h and followed by stimulation with LPS (100 ng/ml) for 6 h. See text for RNA extraction and cDNA analysis by RT-PCR. (B) Microglial cells were treated with SB202190 (5 μM) for 1 h and followed by stimulation with LPS (100 ng/ml) for 6 h. Results are means ± SD from three determinations of a representative experiment which has been repeated 3–4 times. *denotes significant difference, p<0.001.

The observation that p38MAPK activity is not required for stabilization of Nrf2 but is required for induction of HO-1 protein expression suggests that p38MAPK activity may be required for transcriptional activation of HMOX-1 by Nrf2. Thus, steady-state levels of HMOX-1 mRNA were measured in BV-2 cells treated with SB202190. In the first experiment, quercetin (12.5 μM) and SB202190 (10 μM) were added to BV-2 cells one hour prior to stimulation with LPS (100 ng/ml) for 6 h. Following RNA isolation, transcript levels of HMOX-1 were measured by quantitative PCR. In agreement with the protein expression data, transcript levels of HMOX-1 were increased following treatment with either quercetin or LPS alone (Fig 7A and 7B), and the combination of quercetin and LPS induced higher levels of HMOX-1 mRNA expression as compared to levels induced by quercetin or LPS alone (Fig 7A). In the second experiment, we tested whether SB202190 could inhibit HMOX-1 expression in cells treated with LPS (100 ng/ml). Results show that SB202190 (5 μM) markedly reduced HMOX-1 mRNA expression induced by LPS (Fig 7B). Taken together, these results are consistent with the protein results suggesting that p38MAPK activity is required for Nrf2-dependent transcriptional activation of the HMOX-1 gene.

### Assessing the role of quercetin and HO-1 on LPS induced NO production

**Fig 8:**
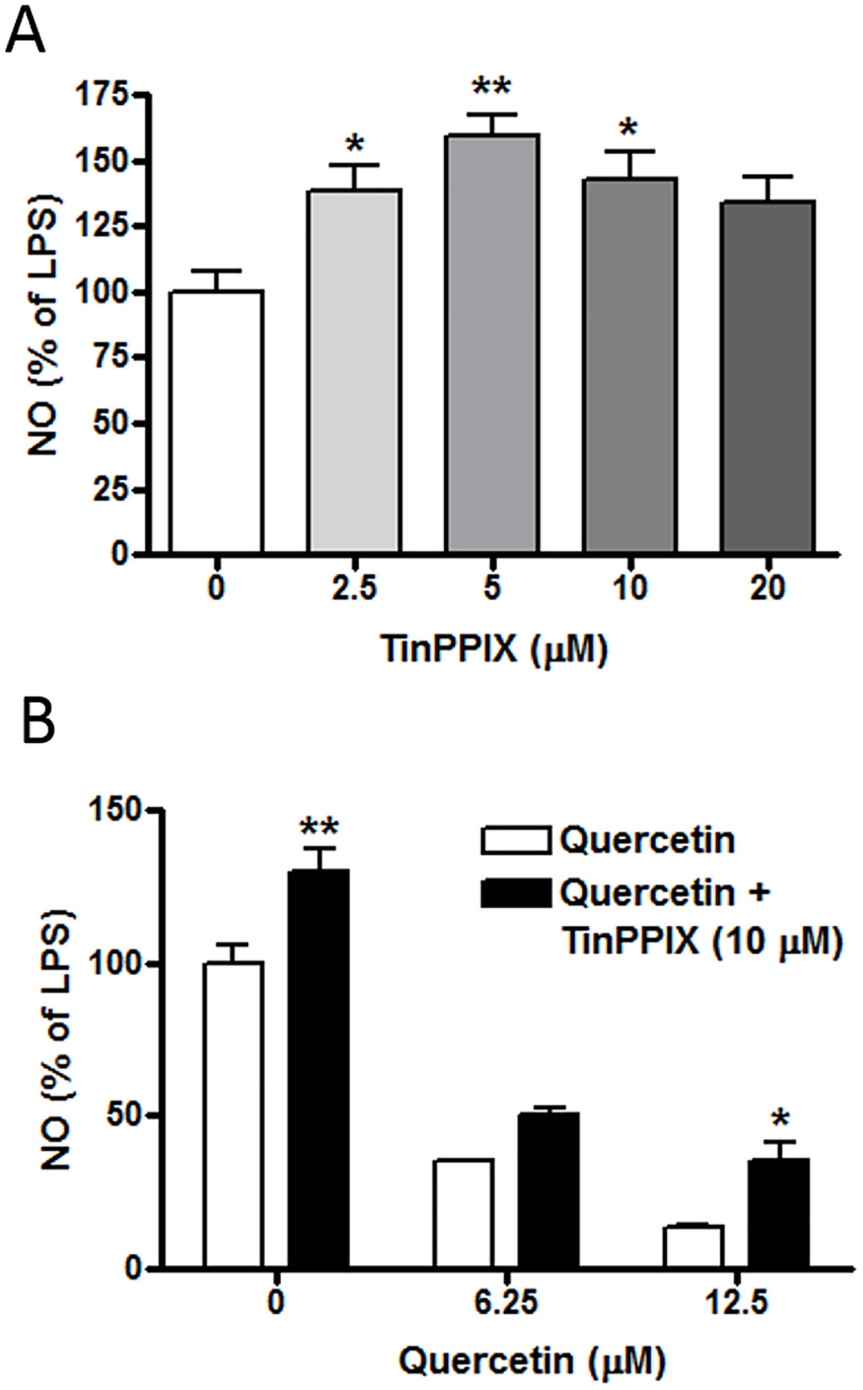
Effects of TinPPIX on LPS- and quercetin-induced NO production. (A) Cells were pretreated with different doses of TinPPIX for 1 h followed by stimulation with LPS (100 ng/ml) for 16 h. Results are means ± SEM from four independent experiments and were analyzed by one-way ANOVA followed by Bonferroni post-tests. *p<0.05, **p<0.01 vs. LPS alone. (B) Cells were treated with different doses of quercetin in the presence and absence of TinPPIX (10 μM) for 1 h followed by stimulation with LPS (100 ng/ml) for 16 h. Results are means ± SEM from three independent experiments and were analyzed by two-way ANOVA with Bonferroni post-tests. *p<0.05, **p<0.01 vs. no TinPPIX.

The results presented thus far demonstrate that treatment of LPS-stimulated cells with quercetin is able to reduce levels of NO production as well as increase levels of HO-1 protein expression. To demonstrate a direct link between HO-1 protein expression and the level of LPS-induced NO production, experiments were carried out with TinPPIX, an inhibitor for HO-1. As shown in Fig 8A, NO production by LPS was increased upon addition of TinPPIX (2.5 to 10 μM), indicating that HO-1 activity reduces NO production in LPS-stimulated cells. In a second experiment, cells were stimulated with LPS in the absence or presence of increasing dose of quercetin, and in parallel, the cells were treated with TinPPIX (10 μM). Consistent with the results described in Fig 1B, quercetin markedly reduced NO production in LPS-stimulated cells. Furthermore, consistent with the notion that HO-1 activity contributes to reduction of NO production, higher levels of NO production were observed in cells treated with TinPPIX (Fig 8B). However, TinPPIX treatment did not fully reverse the inhibitory effect of quercetin (Fig 8B).

## Discussion

**Fig 9:**
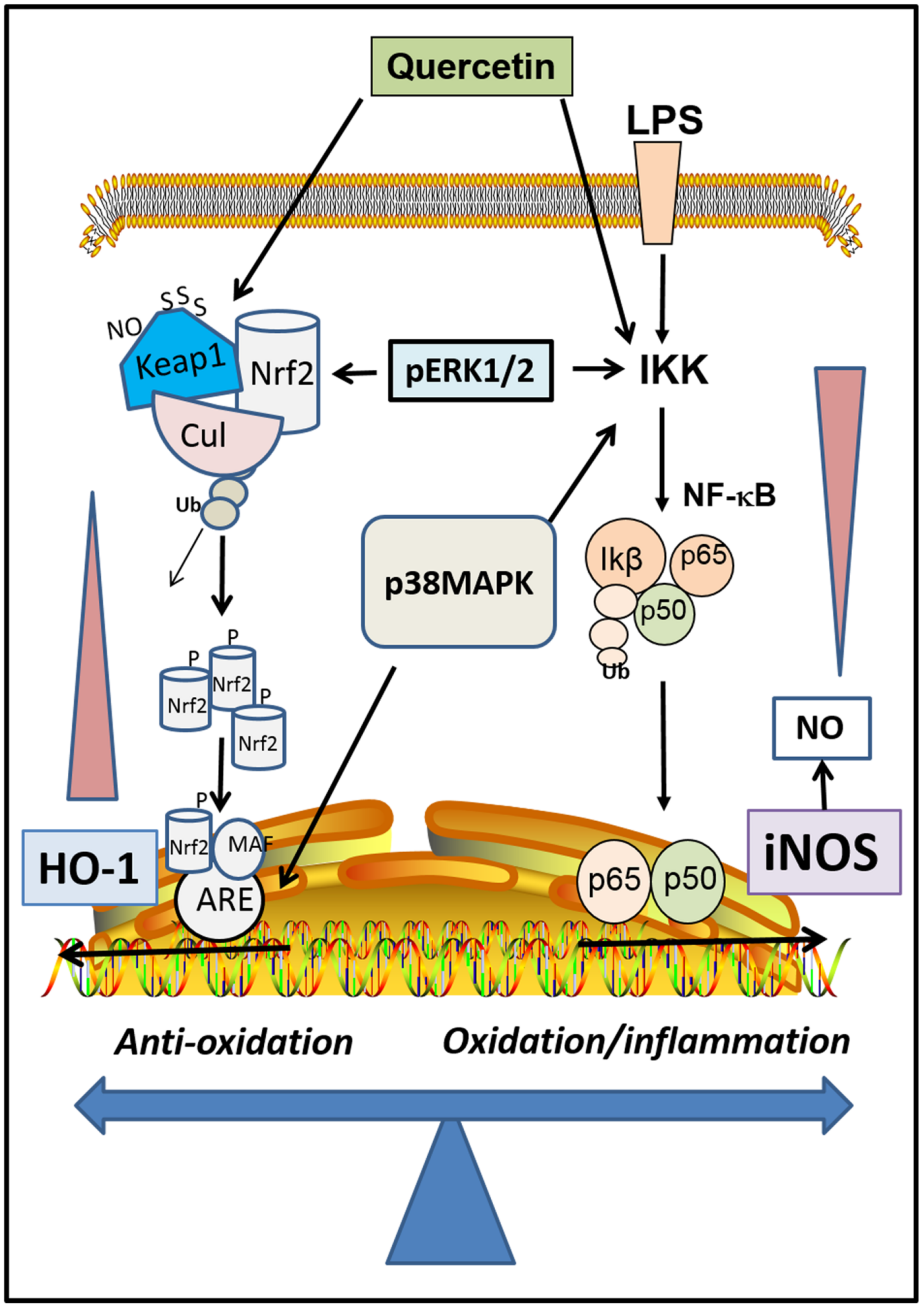
A scheme depicting effects of quercetin upregulating the Nrf2 and down- regulating the NF-κB pathways and possible cross talk with ERK1/2 and p38MAPK.

Quercetin is a flavonol present in berries as well as other fruits and vegetables, including red onion [37]. Recent studies, including those of our own, demonstrated that quercetin potently inhibits LPS-induced ROS and NO production in microglial cells [4,5,38]. In fact, the numerous health effects of quercetin have provided strong support for this dietary flavonol to be an adaptogen and a nutraceutical [39,40]. Besides inhibition of LPS-induced NO, quercetin also could stimulate the Keap1/Nrf2 anti-oxidant signaling pathway, leading to activation of the ARE-regulated genes such as HO-1 [5,26]. The up-down regulation of quercetin on the NF-κB and Nrf2 pathways has generated considerable interest in search for mechanism(s) responsible for the cross talk (Fig 9). In the present study, a comparison between quercetin and cyanidin showed distinct differences on their ability to inhibit LPS-induced NO production, and these differences reflected also in their ability to stimulate Nrf2-induced increased HO-1 protein expression. The differences in effects between these two molecules is partly due to the presence of alpha, beta unsaturated carbonyl in quercetin which facilitates transfer of electrons and thus qualified as an electrolyte (Fig 1). The inability of cyanidin to activate the Nrf2 pathway has been observed in previous studies with macrophages [41] and human keratinocytes [42]. However, these results may also be cell type specific and whether the compound is glycosylated, as cyanidin-3-O-glucoside was shown to activate the Nrf2 pathway and increase HO-1 protein expression in endothelial cells [43]. Many food polyphenols (probably those with electrophilic property) can modulate the Nrf2 pathway by reacting with one or more cysteine residues in Keap1 [13]. Based on the differences between quercetin and cyanidin, it is reasonable to hypothesize that redox active compounds that are effective in inhibiting LPS-induced NO can also stimulate the Keap1/Nrf2/HO-1 pathway (Fig 9).

Our results unveiled another interesting phenomenon showing that while quercetin can itself stimulate Nrf2 and HO-1 production in untreated BV-2 cells, this activity was greatly enhanced when cells were stimulated with LPS. Ability for quercetin to stimulate Nrf2 and HO-1 in LPS-treated cells has been observed previously [26]. Our study demonstrated this effect can be observed at low doses of quercetin (2.5 μM) (Fig 3). Since LPS is an endotoxin known to stimulate oxidative/nitrosative and inflammatory responses through the NF-κB transcriptional pathway [5], it is somewhat surprising that by itself, LPS can also cause the increase in Nrf2 and HO-1 (Fig 2). Consequently, it can be concluded that ability to up-down regulation of the NF-κB and Nrf2 pathways in these cells depends on the levels of botanicals and toxin present, and working in a concerted manner [44]. Besides quercetin, there is evidence that other phytochemicals (probably those with electrophilic properties) also show similar adaptive responses by up-regulating the antioxidant pathway involving Nrf2 and production of anti-oxidative proteins [33,45]. However, more studies are needed to demonstrate the up-down hypothesis and whether these other phytochemicals also show similar exaggerated response upon exposure to endotoxins or other stressors.

Protein kinases, including the MAPKs, PKC and PI-3K have been shown to play an important role in regulating oxidative and anti-oxidative signaling pathways in immune cells and in neurodegenerative diseases [46–50]. Our earlier work with BV-2 microglial cells demonstrated the role of p-ERK1/2 in mediating cross talk between the LPS (NF-κB) and IFNγ (JAK-STAT) signaling pathways that lead to transcriptional induction of iNOS. Phenolic botanicals such as honokiol from Magnolia bark, could inhibit IFNγ-induced p-ERK1/2 [1]. In recent studies, there is strong evidence pointing to the role of p38MAPK in regulating inflammatory responses in microglia and macrophages [51,52]. Although previous studies have focused on the inflammatory pathways, much less is known regarding action of p38MAPK on the Nrf2 pathway. Results in the present study indicate that while LPS and quercetin can individually stimulate p38MAPK phosphorylation, quercetin together with LPS resulted in higher levels of phosphorylated p38MAPK as compared to levels induced by quercetin or LPS alone (Fig 5). On the other hand, although LPS could enhance phosphorylation of ERK1/2, LPS plus quercetin did not show additive effects on phosphorylation of ERK1/2. We further used pharmacological inhibitors for p38MAPK and ERK1/2 to test effects of the MAPKs on quercetin-induced Nrf2 and HO-1 expression. Results show that U0126 effectively inhibited quercetin-induction of Nrf2 and HO-1 protein expression, whereas the p38MAPK inhibitor, SB202190, inhibited mainly quercetin-induced HO-1 expression (Fig 6). These results suggest involvement of ERK1/2 activity on stabilization of Nrf2 and its translocation to the nucleus prior to increased transcription of the HO-1 gene, whereas p38MAPK appeared to target mainly transcription of the HO-1 gene and protein. Study with mRNA further supported the specific action of p38MAPK on HO-1 transcription (Fig 7). Although the mechanism whereby p38MAPK targets HO-1 transcription is not well understood, there is increasing recognition for similar involvement with other botanicals, including lutein, dehydrocostuslactone and ethanol extract of Inula helenium L. [35,52–55].

The ability for quercetin to inhibit LPS-induced inflammatory responses (mainly through the NF-κB pathway) and concomitantly increase in Nrf2/HO-1 in immune active cells has generate questions regarding the extent of activity and products produced by the two pathways, and the extent the Nrf2 pathway can counteract the NF-κB pathway. In particular, HO-1 has been regarded as a key molecule in mitigating the inflammatory responses induced by NF-κB [56]. In our study, a relationship between LPS-induced HO-1 and NO production can be observed by using TinPPIX, a specific inhibitor for HO-1activity (Fig 8). A dose study indicated TinPPIX at 5 μM caused a 50% increase in LPS-induced NO production. When the effect of quercetin on LPS-induced NO production was tested in the presence and absence of TinPPIX, presence of TinPPIX consistently provided higher levels of NO although decreasing NO is observed with increasing dose of quercetin. These results further suggest that induction of HO-1 by quercetin may not completely counteract LPS-induced NO production. One possibility is that besides HO-1, quercetin may induce synthesis of other anti-oxidative proteins through the Nrf2/ARE pathway, and thus further contributes to the anti-oxidative effects on LPS-induced iNOS/NO production. In agreement with this notion, quercetin was shown to be a strong inducer of NQO1 expression in hepatocytes [57]. Taken together, these results provide insights that quercetin exerts potent inhibitory effect to mitigate LPS-induced NO, but this effect is only partially contributed by its ability to stimulate Nrf2 and HO-1 activity. Obviously, more studies are needed to investigate these possibilities and cross-talk mechanisms between the two signaling pathways under different conditions in microglial cells.

## Conclusion

Results of this study promote the hypothesis that compounds that potently inhibits LPS-induced NO production also stimulates the Nrf2 anti-oxidative pathway. Quercetin (but not cyanidin) can combat LPS stress by enhancing the Nrf2 pathway and production of HO-1. ERK1/2 and p38MAPK appear to target the Nrf2/HO-1 pathway at different sites. Finally, quercetin exerts potent inhibitory effect to mitigate LPS-induced NO, but this effect is only partially contributed by its ability to stimulate Nrf2 and HO-1 activity.
